# Enhancing Examination Success: the Cumulative Benefits of Self-Assessment Questions and Virtual Patient Cases

**DOI:** 10.1007/s40670-022-01568-z

**Published:** 2022-08-04

**Authors:** Martha P. Seagrave, Lynn Foster-Johnson, John B. Waits, Katherine Margo, Shou Ling Leong

**Affiliations:** 1 Department of Family Medicine, Robert Larner MD College of Medicine, at the University of Vermont, Burlington, VT USA; 2 Department of Medical Education and The Dartmouth Institute, Geisel School of Medicine at Dartmouth, Hanover, NH USA; 3 Cahaba-UAB Family Medicine Residency, Cahaba Medical Care Foundation, Centreville, AB USA; 4 Family Medicine and Community Health, Perelman School of Medicine, University of Pennsylvania, Philadelphia, PA USA; 5 Department of Family and Community Medicine, Pennsylvania State University College of Medicine, Hershey, PA USA

**Keywords:** Teaching and learning, Assessment, Self-assessment, Medical education, Family medicine

## Abstract

**Purpose:**

Research on the learning benefits of the feedback-rich formative assessment environment of virtual patient cases (VPCs) has largely been limited to single institutions and focused on discrete clinical skills or topical knowledge. To augment current understanding, we designed a multi-institutional study to explore the distinct and cumulative effects of VPC formative assessments and optional self-assessment questions (SAQs) on exam performance.

**Method:**

In this correlational study, we examined the records of 1,692 students on their family medicine (FM) clerkship at 20 medical schools during the 2014–2015 academic year. Schools utilized an established online curriculum, which included family medicine VPCs, embedded formative assessments, context-rich SAQs corresponding with each VPC, and an associated comprehensive family medicine exam. We used mixed-effects modeling to relate the student VPC composite formative assessment score, SAQ completion, and SAQ performance to students’ scores on the FM final examination.

**Results:**

Students scored higher on the final exam when they performed better on the VPC formative assessments, completed associated SAQs, and scored higher on those SAQs. Students’ SAQ completion enhanced examination performance above that explained by engagement with the VPC formative assessments alone.

**Conclusions:**

This large-scale, multi-institutional study furthers the body of research on the effect of formative assessments associated with VPCs on exam performance and demonstrates the added benefit of optional associated SAQs. Findings highlight opportunities for future work on the broader impact of formative assessments for learning, exploring the benefits of integrating VPCs and SAQs, and documenting effects on clinical performance and summative exam scores.

## Introduction

A feedback-rich instructional environment is linked to positive learning outcomes. Much about current learning science relates to feedback. In their seminal work, Brown, Roediger, and McDaniel [1] posit that learning is deeper and more durable when it involves effortful retrieval through interleaving and includes generation, elaboration, and reflection on the material through frequent low-stakes assessment. Using a wide variety of assessment formats necessitates retrieval of knowledge in different ways, incorporates multiple levels of learning, and forces the learner to make connections across different silos of knowledge domains.

Over the past several decades, medical educators have been re-thinking the role of assessment in the classroom. Approaches such as Assessment for Learning [AfL, 2–4], the concept that assessment should be embedded in the learning process to gauge progress, and Test-Enhanced Learning [TEL, 5, 6], the utilization of frequent low-stakes tests to reinforce long-term retention, extend formative assessment techniques to classroom instructional practices [7]. Their focus is on the quality of the learning process and deepening student learning [8]. In these approaches, feedback to support current and future learning is continuously provided [9]. Students are expected to be active participants in their own learning through self-evaluation, self-assessment, and self-regulation [10–12].

In line with the work of Brown et al. [1], both TEL and AfL consider assessments as opportunities to directly boost learning and provide evidence that the deliberate recall of information results in advanced learning, retention, and transfer over repetitive study. A recent meta-analysis of TEL studies found that positive outcomes were robust across test question format, learner type, and health profession discipline [13]. Meta-analytic results indicate that the most effective applications of TEL include repeated testing, the use of context-rich multiple-choice questions (MCQs) that require the application of knowledge, and provision of feedback on both incorrect and correct answers. The success of TEL has been demonstrated for improving clinical reasoning [14], diagnostic accuracy [15], and biomedical knowledge [6]. AfL is also widely used in medical school programs [16] in conjunction with OSCEs in pre-clerkship settings [17] and with learners in clinical settings [9, 18].

Student ownership of their learning and self-regulation is central to these novel applications of assessment. Effortful retrieval and self-evaluation of performance in relation to the outcomes criteria elicit higher-order thinking and meta-cognition. Within medical education, most studies examining the effectiveness of self-assessment have focused on whether performance on formative question banks or quizzes predicts performance on high-stakes examinations such as the *United States Medical Licensing Examination* (USMLE) Step examinations. Zahn et al. [19] found that performance on the National Board of Medical Examiners (NBME) clinical subject examinations in core clerkships was positively correlated with scores on the USMLE Step Examinations. Others have established that scores on the NBME Comprehensive Basic Science and Clinical Science Self-Assessments predict performance on both the USMLE Step Examinations [20, 21] and the NBME Clinical Science Subject Examinations [22]. Moroz et al. [23] showed that increases in scores on the self-assessment examinations in physical medicine and rehabilitation were significantly related to improvements in board scores. Nguyen et al. [24] report that students who took a comprehensive family medicine exam at the beginning of the clerkship and received educational support based on the exam results significantly reduced their NBME subject exam failure rate.

The individualized nature, diverse assessment formats, and real-time feedback inherent to the virtual patient case (VPC) platform make it an ideal instructional approach for promoting students as agents of their own learning. Meta-analytic evidence has shown a clear positive effect for using VPCs as an additive resource, [25–27] and a growing body of work links VPC use to improved learning outcomes [28–32]. Medical students are increasingly using VPC programs as learning aids because of the retrieval practice spaced over time, holistic overview of material, and application of knowledge through low-stakes, formative assessments [33]. The variety of assessment types (e.g., multiple-choice questions, summary statements, clinical reasoning tools) aimed at different levels of learning allows for reflection, elaboration, and generative knowledge retrieval, which strengthens understanding. Students complete the material at their own pace, engage in self-assessment, and may proactively design a learning plan to bolster knowledge gaps identified by the formative assessments in the VPC.

Published studies using VPCs found self-assessment resulted in higher examination scores [34] and decreased failure rates on the NBME examination [24]. Still, there has been little work demonstrating the effectiveness of the feedback-rich formative assessment environment of VPC on subsequent performance on summative assessments in the form of final exams. Most investigations have been single-institution studies, with content limited to discrete clinical skills or knowledge of a single topic, limiting the generalizability of findings. We also found no studies that conceptualized the VPC formative assessments as mechanisms for learning or demonstrated the incremental benefits of self-assessment strategies in andragogy. This gap provided the opportunity for a more comprehensive study. We included a larger sample of medical schools and designed our study to demonstrate the relative contribution of self-assessment to learning, over and above the engagement with VPC formative assessment and feedback. We are using the term andragogy as advanced by Knowles [35] as it focuses on self-directed adult learning rather than more teacher-centric learning principles of pedagogy.

The purpose of this study was to determine the effectiveness of a feedback-rich assessment environment on learning. We posited that greater engagement with formative assessments within a family medicine VPC environment would result in higher scores on a summative final exam. As evidence of students’ enhanced learning self-regulation, we anticipated more robust exam performance for those students who also completed the optional corresponding self-assessment questions in each case.

## Materials and Methods

### Design, Data Source, and Participants

We utilized a retrospective correlational design on VPC use and self-assessment question (SAQ) data extracted from a family medicine virtual patient learning platform and linked that data to the corresponding final examination scores from the associated, separate, examination database. The resulting dataset consisted of information from 1,692 students on their family medicine clerkships at 20 US allopathic medical schools accredited by the LCME.

The source of VPC use and SAQ data was Aquifer (formerly MedU), a non-profit organization that produces the online family medicine course, which comprises 40 VPCs and 4–5 SAQs corresponding to each case. The VPCs are simulated patient encounters covering the learning objectives of the Society of Teachers of Family Medicine (STFM) National Clerkship Curriculum. Case content includes acute, chronic, and health maintenance patient presentations. The students progress through cases directing the clinical process, developing a differential, documenting clinical reasoning, and devising treatment plans. They respond to embedded multiple-choice questions (MCQ) and prompts for clinical reasoning, such as summary statements and differential diagnoses, throughout the case and receive immediate feedback on whether their answers are correct. As a topic is presented, evidence-based information is provided through hyperlinks to “expert content.” In addition, a dynamically generated “engagement” indicator tracks student’s use and performance on the formative assessments throughout the cases, providing continuous visual feedback corresponding to low (red), moderate (yellow), or high (green) levels. Optional case-based NBME style SAQs, which were specifically developed to complement the case material, are revealed at the end of each case, providing students with the opportunity to independently assess mastery of VPC material and apply it to new clinical scenarios.

### Measures

#### VPC Formative Assessment and Feedback

We conceived of the formative assessment and feedback components in the VPC as learning opportunities measured using a variety of item formats. Because of the high correlations between these formative assessment components, we created a composite *Assessment and Feedback Score* comprised of the equally weighted average of the scores on the integrated MCQ, the summary statements, and clinical reasoning toolbar, as well as the amount of time spent on the case [36]. The scoring of these formative assessments occurs iteratively throughout the case. As a student progresses through the case, the percent of correct MCQ responses is calculated. A binary score for the summary statement is assigned by machine learning software and scored as correct if the text in the summary statement demonstrates a 50% or greater correlation with case content. The clinical reasoning (CR) score is a count of text entries, deletions, and changes in the rank order of the differential diagnoses. The total CR score is the larger number between the percent of actions, or the total number of screens viewed. The time score is the percent of screens viewed for 15 s or more. At the end of each case, a final composite *Assessment and Feedback Score* is calculated.

At the time of the study, feedback on the student’s assessment “engagement” was provided as a visual indicator, with red, yellow, or green circles corresponding to low, moderate, or high performance on the composite *Assessment and Feedback Score*. The assessment engagement indicator provided holistic broad-based feedback to the student while they are completing the VPC. Specific feedback on the accuracy of answers is provided for each MCQ.

The composite *Assessment and Feedback Score* is a valid and reliable measure of performance on VPC formative assessments [37]. The confirmatory factor analysis (CFA) on our data provided evidence that the composite *Assessment and Feedback Score* is a unidimensional construct with scores on MCQ, summary statements, clinical reasoning, and time as indicators. The high comparative and normed fit indices (both reaching 0.96) and low standardized root mean square residual (SMR = 0.03) indicate the data fit a one-factor model well [38]. Latent trait reliability is 0.76, and loadings of the four engagement score indicators are positive and statistically significant. We averaged the student’s *Assessment and Feedback Scores* across the completed VPCs and included this score in our model to measure student’s engagement and performance with the formative assessments in the course.

#### Self-Assessment Questions (SAQ)

Four to five optional SAQs are at the end of each VPC and are only accessible after VPC completion. Each question set focuses on the topics addressed in the associated family medicine case. These 197 MCQs are in scenario-based format and written by mentored students, residents, and faculty utilizing a comprehensive question writing guide and reviewed by content experts. Upon completion of a case, students receive detailed explanations of correct and incorrect answer choices.

Analyses of the psychometric qualities of the SAQ items indicate that item difficulty is comparable to that of the family medicine final examination questions [38]. Specifically, the average item difficulty is 0.75, and the mean item discrimination (r_pb_) is 0.26. Our analysis includes students’ average score—the percentage of correct responses for all completed SAQs. A binary variable indicates whether the student completed at least one SAQ corresponding to each of the 40 VPCs.

#### Family Medicine Final Examination

The associated family medicine final examination accompanies the family medicine VPCs and directly aligns with the STFM National Clerkship Curriculum objectives [39]. Trained STFM members developed the final examination questions with a single correct answer. There are two equivalent forms of the examination containing two to three questions corresponding to the content in each of the 40 family medicine VPCs. An annual review of examination psychometrics ensures consistency and quality. For the examination forms used in this study (academic year 2014–2015), the average item discrimination (r_pb_) was 0.20 and 0.22; the mean item difficulty was 0.75 and 0.76, and the coefficient alpha for each form was 0.74 and 0.77 [40].

The Aquifer family medicine examination has established validity, with scores positively correlated with completion of VPCs [28] and the NBME Family Medicine Subject Examination [32]. For our study, we equated examination scores across test forms using linear equating [41], and the total score for each student was included as the outcome variable.

### Analyses

We used descriptive statistics and correlations to examine VPC use and scores on the formative assessments, SAQ completion and scores, and performance on the family medicine final examination. Linear mixed models (LMM) with robust standard errors tested differences in average examination scores and modeled the effect of VPC engagement with assessments and SAQ completion on final examination performance. Four models determined the relative contribution of each factor in explaining final examination performance. The base model (M1) included VPC completion and the *Assessment and Feedback Score*. Model 2 adds the number of VPCs with at least one completed SAQ, and Model 3 contains performance about these SAQs. The final model, M4, included all variables in Models 1–3 and added the time spent on SAQs. We present standardized estimates and pseudo *R*^2^ [42] for each model.

We nested students within their schools to account for the correlated errors associated with clustering. The VPC formative *Assessment and Feedback Score* and the SAQ measures were included in the model as fixed and random effects, and person-level intercepts were included as random effects. All analyses used SAS Version 9.4 for Windows (SAS Institute Inc., Cary, North Carolina). Pseudo *R*^2^ assessed model fit.

## Results

The final sample consisted of 1,692 students on their family medicine clerkship at 20 medical schools during AY 2014–2015. On average, students completed 31.4 of the 40 family medicine VP cases. Thirty-two percent of the students completed all 40 cases in the course.

While completing the family medicine cases, students demonstrated moderate performance, with an average *Formative Assessment and Feedback* score of 65.7. Most students (90%) completed at least one corresponding SAQ for over half of the 40 VPC ($\overline{x }$=27.8) and evidenced content mastery on SAQs with an average score of 75.7%. See Tables 1 and 2.

**Table 1:** Variable definitions and descriptive statistics. Descriptive statistics on study variables for 1,692 students at 20 medical schools

Variable	Mean*	Std. Dev	Min	Max	Definition
Percent of students who completed all VPCs	32%	47%	0%	100%	Percent of students who completed all 40 virtual patient cases (VPCs) in the family medicine course
Number of VPCs completed	31.43	11.22	1	40	Number of family medicine VPC completed (out of 40)
Average *Formative Assessment and Feedback Score* on VPC in family medicine course	65.66	14.37	3.3	100	Composite score comprised of 4 equally weighted components: percent correct on case questions; summary statement score, use of the clinical reasoning toolbar, time spent on each screen
Percent of students who completed at least one SAQ	90%	30%	0%	100%	Percent of students who completed at least one corresponding self-assessment question (SAQ) item for family medicine VPC
Score on final exam	73.8%	10.0%	36%	96%	Score on final associated family medicine exam (percent correct)

**Table 2:** Variable definitions and descriptive statistics. Descriptive statistics on study variables for 1,518 students at 20 medical schools completing self-assessment questions (SAQs)

Variable	Mean*	Std. Dev	Min	Max	Definition
Percent of students who completed all VPCs	34%	47%	0%	100%	Percent of students who completed all 40 Virtual Patient Cases (VPC) in the family medicine course
Average *Formative Assessment and Feedback Score* on VPC in family medicine course	65.71	14.35	4.80	95.0	Composite score comprised of 4 equally weighted components: percent correct on case questions; summary statement score, use of the clinical reasoning toolbar, time spent on each screen
Number of VPCs with at least one completed SAQ	27.75	13.56	1	40	Number of family medicine VPC out of 40 where the student completed at least one corresponding SAQ item
Average score on SAQ items	75.7%	12.2%	0%	100%	Average percentage of correct answers on SAQ items
Average number of minutes spent on SAQ	1.59	0.78	0.09	4.96	Number of minutes spent completing SAQ
Score on final exam	74.2%	9.9%	36%	96%	Score on final exam (percent correct)

^*^Unweighted mean

### Associations Between VPC Use, SAQs, and Examination Scores

Students who completed more VPCs and performed better on the VPC *Formative Assessment* earned higher scores on their final examination (*r* = 0.33 and *r* = 0.16, *p* < 0.001, respectively). Completion of at least one SAQ was associated with higher examination scores (*r* = 0.13, *p* < 0.001, Table 3). For those students who did SAQs, completing at least one SAQ in numerous VPCs was linked to higher examination scores (*r* = 0.27, *p* < 0.001). Better performance on SAQs led to higher final examination scores (*r* = 0.29, *p* < 0.001). Greater amounts of time spent on SAQs was associated with lower SAQ scores (*r* =  − 0.10, *p* < 0.001) and lower exam scores (*r* =  − 0.07, *p* < 0.01), although the correlation indices were low. See Table 4.

**Table 3:** Correlations between final exam scores, family medicine virtual patient case (VPC) assessments, and self-assessment question (SAQ) use. Correlations on study variables for 1,692 students at 20 medical schools^a^

Variables	1	2	3	4
1. Average final exam score	–			
2. Number of family medicine VPC completed	0.33^***^	–		
3. Completed all cases in family medicine course (1 = yes)	0.20^***^	0.52^***^	–	
4. Average *Formative Assessment and Feedback Score* on VPC	0.16^***^	0.10^***^	0.08^***^	*(.76)*
5. Completed at least one SAQ	0.13^***^	0.28^***^	0.13^***^	0.01

^a^*n* = 1,692, Cronbach’s alpha in diagonal for *Formative Assessment and Feedback*

†*p* < .10; ^*^p < .05; ^**^p < .01; ^***^p < .001

**Table 4:** Correlations between final exam scores, family medicine virtual patient case (VPC) assessments, and self-assessment question (SAQ) use. Correlations on study variables for 1,518 students at 20 medical schools who completed VPC and at least one SAQ ^a^

Variables	1	2	3	4	5	6
1. Average final exam score	–					
2. Number of family medicine VPCs completed	0.30^***^	–				
3. Completed all cases in family medicine course (1 = yes)	0.18^***^	0.52^***^	–			
4. Average *Formative Assessment and Feedback Score* on VPC	0.17^***^	0.09^***^	0.08^**^	*(.77)*		
5. Number of VPCs with at least one completed SAQ item	0.27^***^	0.63^***^	0.34^***^	0.06^*^	–	
6. Average score on SAQ items	0.29^***^	0.11^***^	0.10^***^	0.15^***^	0.22^***^	–
7. Average time spent on SAQ items (in minutes)	− 0.07^**^	− 0.01	− 0.04†	− 0.05^*^	0.01	− 0.10^***^

^a^*n* = 1,518. Cronbach’s alpha in diagonal for *Formative Assessment and Feedback*

†*p* < .10, ^*^p < .05, ^**^p < .01, ^***^p < .001

### Effect of SAQ on Final Examination Scores

**Fig. 1:**
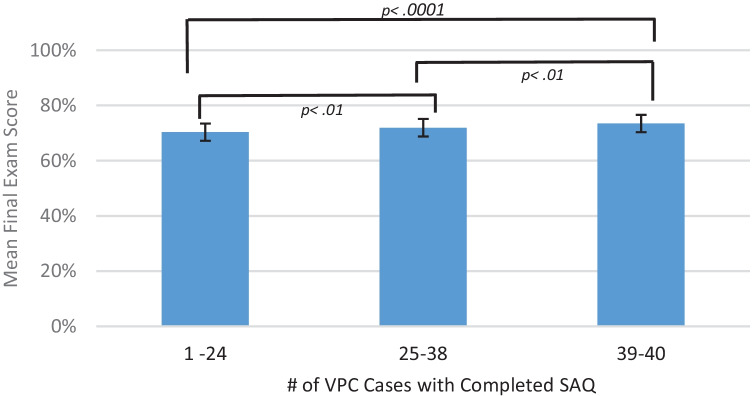
Mean final exam score (percent correct adjusting for mean *Formative Assessment and Feedback Score*), 95% confidence intervals, and post hoc contrasts with Tukey–Kramer HSD test by number of VPC with at least one completed SAQ for 1,518 students at 20 medical schools

A comparison of average scores on the final examination by SAQ completion found significant differences. Controlling for VPC formative assessment performance, students completing at least one SAQ earned significantly higher final examination scores than those not completing SAQs ($\overline{x }$=72.8 *vs.* 70.9, *p* = 0.007). For students completing SAQs, doing more SAQs resulted in significantly higher final examination scores (see Fig. 1).

Using four multilevel regression models, linear mixed modeling (LMM) showed that VPC formative assessment scores and SAQ use explained a significant amount of variance in subsequent final examination performance (see Table 5). In the first model, VPC *Formative Assessment and Feedback* score and completing all VPCs in the course were strong, significant predictors of examination performance, accounting for over 9% of the explanatory variance in examination scores. The addition of SAQ completion to the model increased model *R*^2^ by 14%, explaining 23% of the variance in examination performance (Model 2). Adding SAQ scores to Model 3 significantly improved model *R*^2^ by 3%, accounting for 26% of examination performance. The addition of time spent on SAQ (Model 4) did not improve the model.

**Table 5:** Linear mixed models of VPC use, formative assessments, and SAQ use explaining scores on family medicine final exam

	Model 1			Model 2			Model 3			Model 4	
	β	SE		β	SE		β	SE		β	SE
Intercept	− 0.139	0.126		− 0.130	0.116		− 0.124	0.114		− 0.122	0.114
Completed all VPCs in course (1 = yes)	0.080	0.025^**^		0.052	0.021^*^		0.051	0.020^*^		0.049	0.020^*^
*Formative Assessment and Feedback Score* on VPC	0.180	0.033^***^		0.172	0.031^***^		0.131	0.024^***^		0.132	0.024^***^
Number of VPC with at least one completed SAQ				0.136	0.037^***^		0.106	0.035^**^		0.108	0.035^**^
SAQ score							0.206	0.034^***^		0.203	0.035^***^
Time spent on SAQ (minutes)										− 0.029	0.025
Model fit											
-2 Res Log Likelihood (smaller is better)	3,884.2			3,856.8			3,793.8			3,796.4	
AIC (smaller is better)	3,892.2			3,864.8			3,803.8			3,808.4	
Pseudo *R*^2^	.093			.231			.263			.261	
Δ*R*^2^				.138 *(M2-M1)*			.032 *(M3-M2)*			− .001 *(M4-M3)*	

Linear mixed models of VPC formative assessment and SAQ use explaining scores on family medicine exam for 1,518 students at 20 medical schools who completed VPC and at least one SAQ

*n* = 1,518; ^*^p < .05; ^**^p < .01; ^***^p < .001

Overall, scores on SAQs were essential in explaining performance on final examinations (*β* = 0.21, *p* < 0.001, Model 3). Performance on formative assessments within the VPCs and the number of SAQs completed were similarly effective in increasing final examination scores (*β* = 0.13 and 0.11, respectively). The amount of time spent on SAQs (*β* =  − 0.03) did not reach statistical significance.

## Discussion

This large multi-institutional study provides evidence that student VPC use and VPC engagement with formative assessments positively affect performance on a comprehensive summative subject examination. It further demonstrates that utilization of associated self-assessment questions results in higher scores on the family medicine final examination, beyond that gained from the VPC formative assessments alone. Our findings confirm and extend prior research documenting the benefits of VPCs and SAQs on performance on summative examinations [20, 22].

Cutrer and his colleagues posit that self-assessment promotes the adaptive learner by compelling students to recall knowledge learned in one context and apply it to an alternate scenario [43]. It makes sense that if we provide students with a framework of knowledge in the clinical context and then challenge them with alternative assessment opportunities requiring application or transfer of knowledge, they will demonstrate enhanced comprehension and retention. We used a structured learning platform for this study, which allowed us to track student formative assessments, effort toward self-assessment, and levels of critical thinking and adaptive learning with student progression through the VPCs and SAQs. Providing such self-assessment opportunities increases knowledge acquisition and allows students to engage in intentional metacognitive strategies and self-reflection to enhance their learning [44–46]. Our study corroborates this premise and suggests that the role of VPCs and SAQs goes beyond basic knowledge acquisition. Our approach supports the metacognitive process, assisting the student in identifying learning gaps and promoting critical thinking through applying acquired knowledge to unique scenarios.

There are several strengths to this study. We studied a large cohort of learners across numerous medical schools and utilized a deliberate approach to investigate the issue. We used a comprehensive family medicine curriculum with VPCs and content-related SAQs in conjunction with a final examination aligned with the VPCs. This provided a unified and logical progression from knowledge acquisition to self-assessment and then summative evaluation through the comprehensive examination.

In addition, our measures possess strong theoretical grounding and psychometrics. Student engagement with formative assessment is usually based on self-report. For our study, we captured engagement with the formative assessment using a composite measure comprised of performance metrics within the VPC program [36]. SAQ items are at a similar level of difficulty to final examination items, ensuring equivalence. Separate measures of completion and performance on SAQs allow us to ascertain the incremental increase in examination performance due to self-assessment activities. This provides the opportunity to link VPC and SAQ use metrics to outcomes directly. The enhanced detail of our approach yields greater confidence that this progressive integrated andragogy, which employs a “teach, ask, apply” process for educating medical students, has a notable impact on developing clinical reasoning and knowledge retention.

Several limitations warrant mention. The relatively small model variance (pseudo *R*^2^ = 0.26) suggests that other factors contribute to students’ performance on comprehensive examinations, and we were unable to account for student preparedness and motivation. We also know that the method of integration of VPCs with medical school curricula affects learning outcomes [47], for example, whether VPCs are discretionary activities or a mandatory requirement impacts outcomes such as the number of VPCs completed, scores on NBME subject examinations, and summative clinical ratings [48].

As with other work documenting the impact of VPCs, TEL, and AfL on learning [26, 27], the effect sizes in our study were relatively small. However, the small effect sizes are typical for medical education research, and the sizeable number of participants at different schools ensures that we have a wide range of student abilities and motivations. Given the comprehensive nature of our study, we are confident in the accuracy and generalizability of our findings.

## Conclusions

This study highlights opportunities for future work to assess the broader impact of formative assessment, engagement and self-assessment in improving clinical performance, and comprehensive licensing exam scores. To further understand andragogy that aids retention and clinical reasoning, we need to identify critical features of effective VPCs and SAQs. Additionally, we can explore approaches for integrating VPCs and SAQs in an active learning environment. Recent studies have begun to examine the use of validated examinations as pretests for NBME subject examinations [24]. This exploration could contribute to developing additional SAQs with the necessary psychometric properties to support students’ desire for study aids that will positively affect examination performance.

This multi-institutional study furthers the body of research on the effect of formative assessments within VPCs and the use of optional self-assessment questions on examination performance. Its unique features include the extensive number of students studied, a solid composite indicator of formative assessment and feedback in an integrated learning environment, and verifiable student performance and progression through the course. The results expand the understanding of the role of self-assessment in learning, leading us to stress the importance of developing and incorporating innovative opportunities that engage the student in the learning process.

## Data Availability

Not applicable.
